# Network Analyses in Plant Pathogens

**DOI:** 10.3389/fmicb.2018.00035

**Published:** 2018-01-30

**Authors:** David Botero, Camilo Alvarado, Adriana Bernal, Giovanna Danies, Silvia Restrepo

**Affiliations:** ^1^Laboratory of Mycology and Plant Pathology (LAMFU), Department of Biological Sciences, Universidad de Los Andes, Bogotá, Colombia; ^2^Grupo de Diseño de Productos y Procesos, Department of Chemical Engineering, Universidad de Los Andes, Bogotá, Colombia; ^3^Grupo de Biología Computacional y Ecología Microbiana, Department of Biological Sciences, Universidad de Los Andes, Bogotá, Colombia; ^4^Laboratory of Molecular Interactions of Agricultural Microbes, LIMMA, Department of Biological Sciences, Universidad de Los Andes, Bogotá, Colombia; ^5^Department of Design, Universidad de Los Andes, Bogotá, Colombia

**Keywords:** networks, bacterial pathogens, plant pathogens, host-pathogen interactions, pathogenicity

## Abstract

Even in the age of big data in Biology, studying the connections between the biological processes and the molecular mechanisms behind them is a challenging task. Systems biology arose as a transversal discipline between biology, chemistry, computer science, mathematics, and physics to facilitate the elucidation of such connections. A scenario, where the application of systems biology constitutes a very powerful tool, is the study of interactions between hosts and pathogens using network approaches. Interactions between pathogenic bacteria and their hosts, both in agricultural and human health contexts are of great interest to researchers worldwide. Large amounts of data have been generated in the last few years within this area of research. However, studies have been relatively limited to simple interactions. This has left great amounts of data that remain to be utilized. Here, we review the main techniques in network analysis and their complementary experimental assays used to investigate bacterial-plant interactions. Other host-pathogen interactions are presented in those cases where few or no examples of plant pathogens exist. Furthermore, we present key results that have been obtained with these techniques and how these can help in the design of new strategies to control bacterial pathogens. The review comprises metabolic simulation, protein-protein interactions, regulatory control of gene expression, host-pathogen modeling, and genome evolution in bacteria. The aim of this review is to offer scientists working on plant-pathogen interactions basic concepts around network biology, as well as an array of techniques that will be useful for a better and more complete interpretation of their data.

## Introduction

Biology has entered a new era of scientific discoveries as a consequence of the development of new technologies, and the production of massive amounts of biological data at the cellular and subcellular levels. Researchers can now formulate new hypotheses and diverse manners of testing them. They can design new experiments based on multiple environmental, temporal, and physiological conditions on a single cell, populations, or communities of species. The reduction in costs of next-generation sequencing (NGS) technologies coupled with the advances in metabolomics and proteomics has made high-throughput data more accessible (Hou et al., 2015). The levels of information that can be obtained from biological entities range from genes, genomes, transcriptomes, proteomes, and metabolomes to phenotypes.

Despite these advances, the amount of collected data is larger than the amount being analyzed. Molecular biologists tend to focus on a single level of information (e.g., specific genes, protein-protein interaction, etc.), ignoring the different levels of interactions and connections present within complex biological systems. In the case of quantitative experiments in the areas of genomics and transcriptomics, the amount of available data exceeds the capacity of the common computational systems as well as the ability for researchers to interpret them. Thus, the challenge resides in building models that accurately represent nature and gaining biological insights from data that is inherently noisy and heterogeneous. Systems biology attempts to bridge this multi-level understanding of living systems (Karr et al., 2012).

Systems biology is a discipline that studies biological entities as a whole. Here, parts of the organism (genes and their regulation, signaling cascades, interacting proteins, structural compounds, and metabolic pathways) interact among them and with the environment (which, in turn, gives a context to the organism) to produce a given phenotype. When the biological parts of an organism are interconnected, new properties arise that are dependent on the context and the biological system. Systems biology uses different sources of biological data, mathematical approaches, and computational methods and techniques, to model the organism in a computer (*in silico*). The computational model allows researchers to make predictions and to generate new hypotheses that may then be experimentally validated. Experimentation can fulfill this function and serve to better parameterize and tune different theoretical models.

One of the fields within systems biology which has been fundamental for studying biological organisms at a large-scale is network analysis. Network analyses are a set of mathematical and computational approaches that may be used to study the interactions between the components of a network such as computers connected through the internet, electrical nodes within a network, or biological components within an organism. In the context of biology, the network approach or network biology allows to reconstruct molecular interactions and uncover biological properties that may be difficult to uncover when studying a single or few interactions.

This review presents the main approaches in network biology and their complementary experimental assays used to investigate bacteria-plant interactions. When necessary, examples of human-pathogen interactions were included to illustrate analyses that may potentially be applied to study plant-pathogen interactions. Pathogenicity is an ecological interaction influenced by many different factors. Understanding molecular and ecological interactions may help explain the mechanisms by which pathogens colonize their host plant as well as the co-evolutionary history among the two or more-interacting species.

The review is divided into five sections. First, we describe the basic concepts in network biology; second, we illustrate the importance of metabolic pathways in bacterial pathogenicity; third, we review different approaches used to study protein-protein interactions; fourth, we review the modeling of regulatory networks; and fifth, we describe how this information, may be used to understand processes of adaptation of pathogens to recent and former hosts. The aim of this review is to offer scientists in the field of host-pathogen interactions, the most important concepts around network biology, as well as an array of techniques that will be useful for a better and more complete interpretation of their data.

## Network analyses in systems biology

Network biology has arisen as a new subfield of systems biology (Box 1) useful in molecular biology studies. The high amounts of data produced by omics technologies nowadays, as well as the increasing number of studies on bacterial pathogenesis allows the use of network biology to mathematically model large-scale bacterial systems. Network biology, is a top-down approach (Box 1) that allows the reconstruction of genome-scale biological systems.

Box 1 **Systems biology.**

Systems biology comprises different combinations of mathematical and computational approaches used with diverse kinds of the biological data; as a result, the starting scale of the model (whether it takes into account a small subsystem or a whole system) will vary. Therefore, systems biology may use two approaches that are complementary depending on the nature of the data, and the mathematical and computational approaches used: **bottom-up** and **top-down** approaches (Bruggeman and Westerhoff, 2007).

The **bottom-up** approach precisely reconstructs biological subsystems from their parts (genes, proteins, and metabolites) until a full model of the subsystem is obtained (mainly at a small scale). This kind of approach allows to **deduce** fundamental principles inherent to all biological systems such as the physical and mathematical laws that govern it. The data used for the model are obtained from single cell experiments, from the *in vitro* assessment of rate parameters from enzymatic reactions, transport phenomena, or regulatory processes.

The **top-down** approach reconstructs the biological system from high amounts of data to initially obtain a full draft model of the whole system, with subsequent refinings. This kind of approach allows to **induce** properties of the system in a biological state. The data used for these models arise from omics experiments (genomics, transcriptomics, metabolomics, etc.), and they allow the reconstruction of the whole model.

The biological networks represent the relationships among molecular components within the context of a cellular function (Box 2). The methods derived from the mathematical framework of networks can be applied to diverse fields such as electrical, social, and Internet networks. As biological systems can be represented as networks, the mathematical concepts behind network analyses can be applied to biomolecular systems.

Box 2 **Biological networks.**

**Figure 1:**
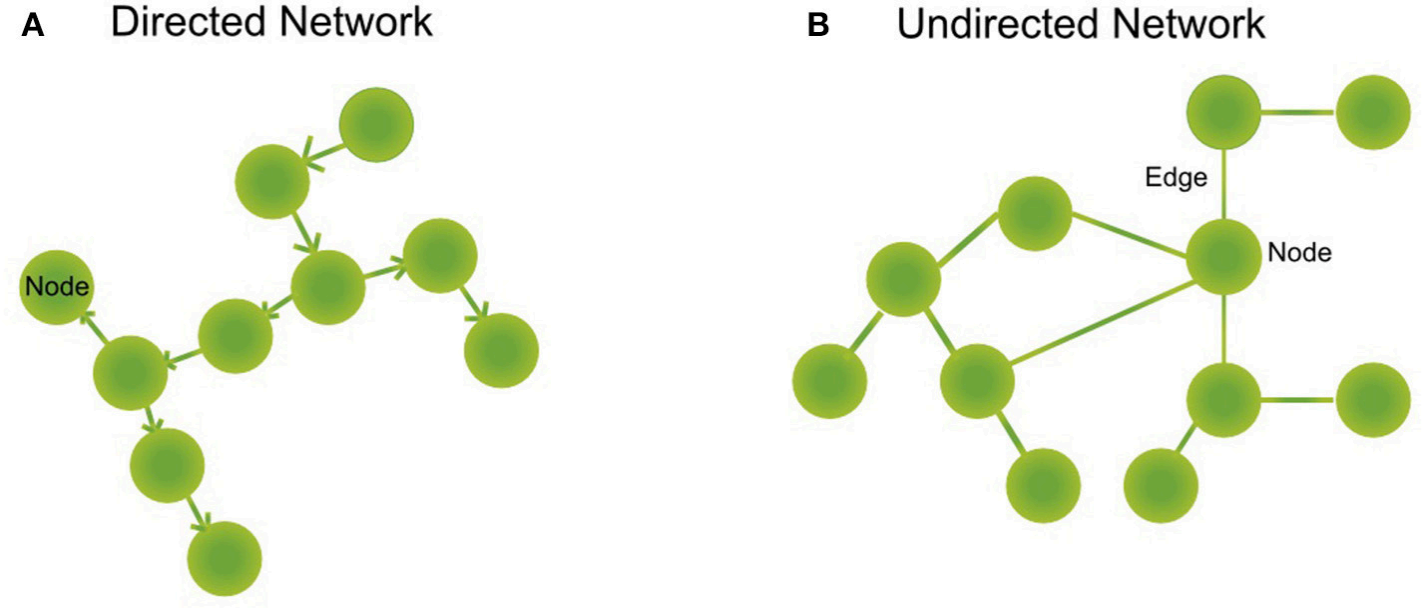
Type of networks. **(A)** Directed networks are composed of nodes representing biological entities as proteins, metabolites, or genes. These nodes are interconnected by directed edges (or arrows) that symbolize a directed relationship between two or more biological species, as a gene regulated by a transcription factor or a reaction that is connected downstream to another reaction forming metabolic pathways. **(B)** Undirected networks are composed of nodes, that represent proteins, for example. These nodes are interconnected by edges that symbolize an interaction between two or more biological species, as for example signaling proteins.

A biological network is a mathematical abstraction of nature which represents biological entities such as genes, transcription factors, metabolites, and proteins, as **nodes** or **vertices** and the relations between them as **edges** or **links** (regulatory mechanism, transformation reactions, protein-protein interactions such as signaling cascades). We can find **directed** (regulation of A to B, directionality of an enzymatic reaction, Figure 1A) or **undirected** (a pair of interacting proteins, Figure 1B) networks, depending on whether the relationship between the nodes has directionality or not, or if this can be determined. Furthermore, there are **unipartite** networks where the nodes have the same biological feature (e.g., protein-protein interaction networks) and **bipartite or two-mode** networks, composed of different biological components (e.g., a regulatory network where regulatory proteins and regulated genes interact or, metabolic networks where substrates are connected to reactions and reactions with substrates) (Newman, 2010).

### Types of biological networks

In the context of networks and molecular biology, we can represent an organism, or parts of it, using four different kinds of networks: regulatory, metabolic, protein-protein interaction networks (PPINs), and signaling networks (a special type of PPIN). Furthermore, these networks can be integrated into a single model by using a combination of different networks connected into a single computational model. It is important to note that the classification of regulatory, metabolic, and PPINs is arbitrary and has been done to facilitate the construction of scientific knowledge. This review, focuses on these three methods due to the availability of omics data from pathogens that may be used to construct these types of networks. The omics data that have been generated have been mostly used to investigate specific research questions, leaving large amounts of data yet to be explored. Network analyses provide an opportunity to further analyze this information to develop new hypotheses related to mechanisms of pathogenesis or general life style of these microorganisms.

One type of network is the transcriptional regulatory network (TRN). TRNs are used to mathematically represent gene expression profiles and their regulation by transcription factors or other regulatory elements (e.g., sRNA). Through these TRNs, one can simulate the effect of different biological and environmental conditions on the expression profile of an individual. The TRNs may be constructed for specific groups of genes, such as those related to pathogenicity, or for the whole organism. In a topological sense, the TRN is defined as a bipartite network (Box 2) with directionality. Some nodes correspond to regulatory proteins and others to target genes (that can be transcriptionally switched on or off by the regulatory protein). One regulatory protein can be connected to several target genes; in turn, genes can be regulated and connected by one or a small number of regulatory proteins.

Metabolic networks are substrate-product transformation networks mediated by enzymatic reactions. In the metabolic networks, the substrates and products can be proteins, lipids, and other cellular components. These are represented as nodes and the transformation reactions mediated by enzymes are represented as edges. This representation of metabolic networks can be analyzed by computational methods to perform associations between the genotype and the metabolic phenotype of an organism, as constraint based modeling does (Box 3). Metabolic networks may be coupled to the regulatory networks of an organism to model a more complex representation of the molecular machinery of the organism.

Box 3 **Systems biology.**

The metabolism of an organism may be represented in a matrix based on the stoichiometry of the reactions in the **constraint-based modeling** (CBM) approach (Orth et al., 2010). The stoichiometric matrix can be analyzed to assess the metabolic phenotype of the organism under different conditions (e.g., environment, mutants, etc.). To analyze the metabolic phenotype, the stoichiometric matrix may be solved using a Flux Balance Analysis (FBA). A FBA is a computational optimization method. The final solution of the metabolic system is the distribution of the reaction rates or fluxes (moles over time). In the FBA, assumptions and constraints of the system are defined. For example, it assumes a steady-state (thermodynamic equilibrium) and defines upper and lower boundary constraints for the fluxes throughout the reactions. Furthermore, an objective function must be defined to achieve a unique solution of the system. The **objective function** is a reaction or a combination of reactions that represent a biological feature of the organism e.g., biomass. In other words, models based on CBM approach represents the metabolism of an organism only with the information of the reactions catalyzed by enzymes that are coded in the genome.

Another alternative approach that does not require the calculation of an optimal flux distribution is the **elementary flux mode analysis** (EFMA) (Zanghellini et al., 2013). In this analysis, the metabolic network is decomposed in its main pathway components.

A complementary analysis in metabolic modeling is **gene set enrichmen**t **analysis** (GSEA) (Hung et al., 2012). When applied to a genome-scale, set of genes differentially expressed can be classified into metabolic categories or pathways giving information related with the most represented pathways in a determined scenario.

A PPIN reflects physical interactions between two or more proteins. In this category, we can find signaling networks, but it is also possible to find proteins involved in the formation of macromolecular complexes related to structural and molecular types of machinery of the cell. The Signaling Network contains a series of proteins that are transformed to carry a signal inside or outside of the cell. Signaling cascades are of special interest in molecular pathosystems since they are tightly related to the regulation of the response to attack and defense of the pathogen and the host, respectively.

When a biological network follows the power law distribution several biological interpretations based on the network metrics can be stated (Box 4). However, these interpretations must be carefully reviewed from the biological point of view of the researcher. We recommend the work of Winterbach et al. (2013), which provides detailed description of these statistics (Winterbach et al., 2013). For a more extensive revision of the mathematical foundations of biological networks, please refer to De Smet and Marchal (2010), Képès (2007), and Newman (2010).

Box 4 **Topological features of biological networks.**

The most basic topological measure of a network is the **average (or mean) degree**, that is the average number of nearest nodes connected to a specific node. More informative is the distribution of these nearest nodes in a network. A feature of some types of biological networks, particularly in metabolic and protein-protein interaction networks is that they follow a **power law** distribution (or a **scale-free** degree distribution). That is, most of the nodes have few neighbors and the minority of them have hundreds or thousands of neighboring nodes (highly connected nodes or **hubs**) (Barabási and Albert, 1999). Particularly the biological networks have a specific topology that tends to be small-world; in other words, their organization makes possible the existence of hubs as central focal points of interactions (Aloy and Russell, 2004), e.g., proteins or genes involved in regulatory processes of bacterial pathogenicity or plant resistance (Haynes et al., 2006).

Another powerful structural measure is the **clustering coefficient**, which evaluates the degree of grouping between a node and its neighbors. The clustering coefficient is defined as the ratio of the number of connections between the neighbors of a node and the total number of possible connections (Watts and Strogatz, 1998). This coefficient measures the **modularity** of a set of nodes and interestingly this feature has shown some patterns of hierarchy between clusters of nodes (modules) of known metabolic networks (Ravasz et al., 2002). Finally, the motifs are patterns of connections between a few nodes that are related to a biological function, particularly in regulatory networks (Maslov and Sneppen, 2002). In topological terms, motifs are sets of nodes whose pattern is overrepresented in the network when compared to a randomly generated network of the same size. In Table 1 we describe other relevant measurements and concepts of networks.

**Table 1:** Basic concepts of biological networks.

**Structure assessment**	**Definition**	**Utility**	**References**
Degree distribution	Distribution of probabilities of degrees in a specific network.	Comparisons, scale-free networks. Clear indicator of the presence of hubs when it is combined with the centrality measurement. Degree provides clues about modules in a network by determining the number of interactions shared between neighboring nodes.	Képès, 2007
Shortest path	The shortest path between two nodes in a biological network.	Connectivity.	Perumal et al., 2009
Average diameter	The minimum number of edges connecting any two nodes over all possible pairs.	Information flow, Small World. Capacity and time of the response of a system, so that in networks with a high centrality, signaling processes are favored.	Képès, 2007
Node clustering coefficient	The ratio of connections to neighboring nodes to the number of all possible connections.	Comparisons, scale-free, hierarchical.	Képès, 2007
Betweenness—centrality	The ratio of the number of k-shortest paths passing through a node and its nearest neighbor links.	Identifies hubs (highly connected nodes in a network), important in pathogenicity and potential target for drugs. Hubs may potentially disconnect the network if they are removed or blocked.	Goh et al., 2001; Perumal et al., 2009
Assortativity	The probability of connection of a node with others of the same degree.	Robustness to node deletion.	Newman, 2010

*Summary of structural measurements of the topology of a network and their utility in a biological context*.

## Metabolic networks and pathogenicity

In this section, we will review studies on metabolic modeling of plant pathogenic bacteria. Given that the information may be limited, we will also include examples of animal pathogens. First, we will describe the constraint-based modeling (CBM) approach, commonly used for *in silico* metabolic modeling. Second, we will review the biological results produced by these studies and their main conclusions; of special interest will be the objective function. Third, we will review the multiscale metabolic modeling approach that integrates different sources of data and constraint-based metabolic models. Finally, we will discuss how CBM is a hypothesis-driven approach used in metabolic networks and the possibilities to improve metabolic models based on experimental results.

### Constraint-based modeling

The metabolic interactions within an organism can be modeled and analyzed using different mathematical approaches, among others, deterministic kinetic models, stochastic models, elementary flux mode analysis, CBM, and pathway enrichment analysis (Box 4; Puchałka and Kierzek, 2004; Hung et al., 2012; Zanghellini et al., 2013). Every one of these methods has advantages and disadvantages. The CBM approach has been established as a standard for metabolic model formulations; there are approximately 165 models of organisms that are finished and experimentally validated (http://sbrg.ucsd.edu/InSilicoOrganisms/OtherOrganisms). This method has been widely employed given that it is a top-down approach (Box 1) that may incorporate whole-genome data and chemical information that is publicly available, as well as knowledge obtained through experimentation into a genome-scale metabolic model. With this approach, several analyses can be performed and relevant biological questions can be addressed (Oberhardt et al., 2009). Other mathematical approaches, such as the mass-action kinetic model (Horn and Jackson, 1972) or the biochemical system analysis (Savageau, 1969), rely on several parameters such as rates of transformation of molecules involved in metabolic reactions of the cell. These parameters are difficult to calculate experimentally at a whole-genome level (that is, all the possible reactions catalyzed by all the enzymes coded by the genome). Thus, the CBM offers a powerful approach to assess metabolic phenotypes in distinct environmental conditions by relying on physicochemical constraints that restrict the metabolic phenotype^1^ of the organism.

**Figure 2:**
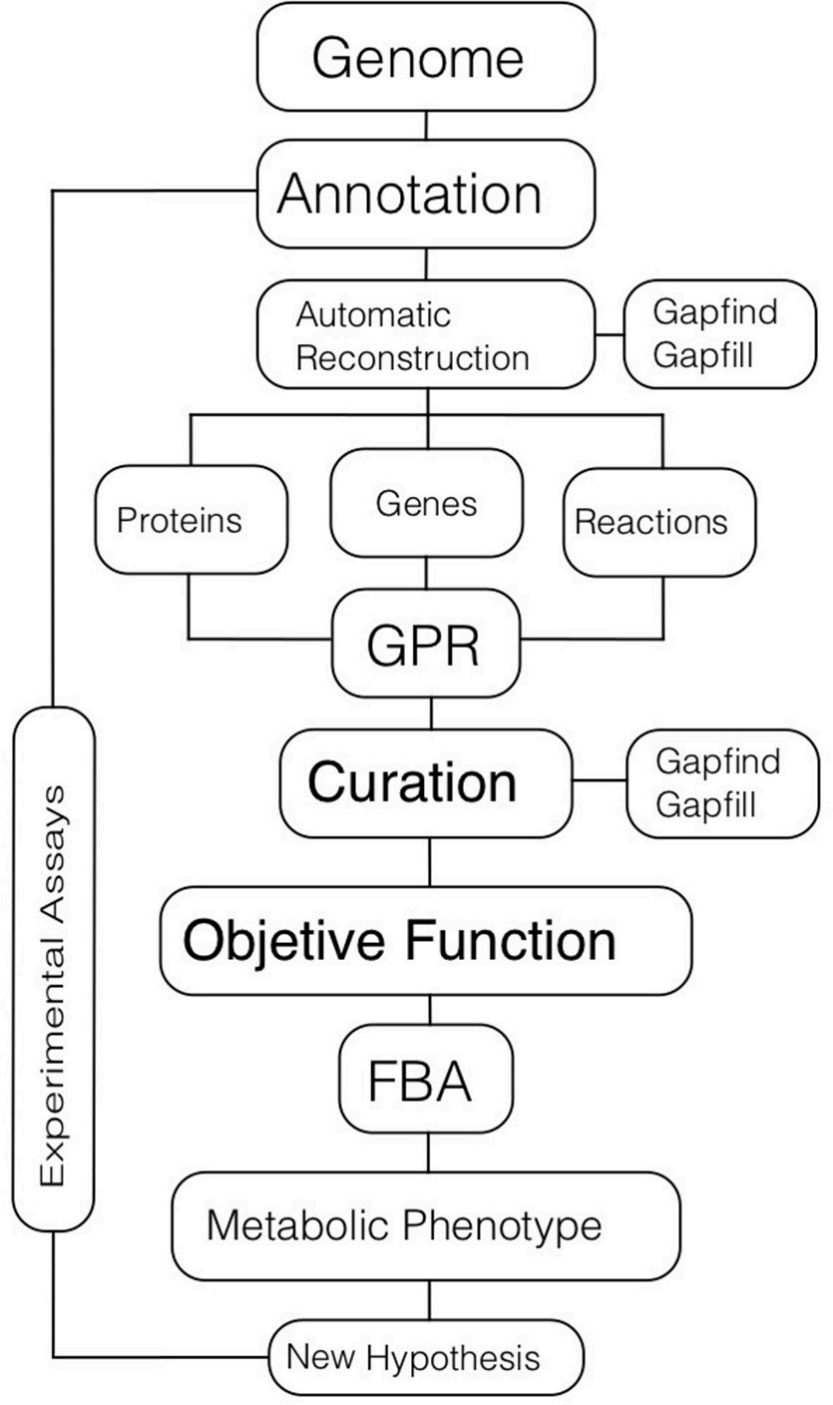
Metabolic modeling. The process of metabolic modeling starts with a genome annotation used for inferring metabolic reactions that are present in an organism. Automatic tools could be used for reconstructing the metabolic network based on the genome. In the initial set of reactions there will be metabolic gaps or missing reactions that are necessary for the complete function of pathways. These gaps can be identified and filled out using different algorithms. The final metabolic reconstruction will have associations among genes, proteins, and reactions (GPRs). Then, further manual curation, based on omics data and literature should be performed. The definition of an objective function that represents a target biological function to optimize should be defined, typically cell growth or ATP production. Once the objective function is set, computational simulations for obtaining metabolic phenotypes related to different conditions are carried out; Flux Balance Analysis (FBA) is the main technique for these simulations. Finally, new biological hypotheses are generated and validated. In all the procedure, data, and information from different experimental assays are incorporated into the model.

The first part of the CBM approach is the genome-scale reconstruction of the metabolic network (Figure 2). There are five main steps in obtaining a high-quality metabolic reconstruction of an organism: (i) genome annotation; (ii) gene-protein and protein-reaction associations; (iii) model curation; (iv) validation through experimental analyses; and (V) improvement of the metabolic model by incorporating the feedback obtained through experimentation.

Genome annotation can be performed using different bioinformatics tools, such as the Rapid Annotation using System Technology (RAST; Aziz et al., 2008; Richardson and Watson, 2013; Kalkatawi et al., 2015). After the genomes have been automatically annotated, they must be manually curated. Once a high-quality genome annotation is obtained, proteins involved in metabolic reactions are assigned. Commonly used databases for the assignment of proteins to metabolic pathways include the Kyoto Encyclopedia of Genes and Genomes (KEGG) (Kanehisa and Goto, 2000), MetaCyc (Caspi et al., 2014), MetaNetx (Ganter et al., 2013), and Biochemical, Genetic and Genomic (BiGG) knowledge base (Schellenberger et al., 2010).

Once the reactions related to the organism of interest are obtained, a mathematical representation of the connected reactions (metabolic models or pathways) can be reconstructed (Orth et al., 2010). However, this initial representation is not free of gaps and errors. These may arise for different reasons such as an inherent gap in our knowledge of bacterial metabolism (e.g., protein-reaction associations), the incomplete genome sequencing of the organisms, or the inaccuracy in the genome annotation. Therefore, the metabolic model needs to be subjected to a curation process. Several methods and algorithms have been developed to curate this model (e.g., based on homology and phylogenetic information or experimental data) (Orth and Palsson, 2010).

Some useful automatic tools that can be alternatively used to reconstruct and analyze metabolic networks include RAST-SEED (Aziz et al., 2008), KEGG Automatic Annotation Server (KAAS) (Moriya et al., 2007), Reconstruction, Analysis, and Visualization of *Metabolic* Networks (RAVEN) (Agren et al., 2013), PRofils pour l'Identification Automatique du Métabolisme (PRIAM) (Claudel-Renard et al., 2003), SuBliMinal, and Pathway Tools (Swainston et al., 2011). Furthermore, protocols for supervised and manual reconstruction of metabolic networks have been established (Francke et al., 2005; Reed et al., 2006; Thiele and Palsson, 2010; Pinzón et al., 2011; Lewis et al., 2012).

Once a representation of the metabolism of the bacterium is obtained, relevant biological questions can be addressed based on this model. For example, the rate of ATP production or the oxygen consumption can be assessed. In the CBM approach, several constraints are set to assess the metabolic model of the organism (McCloskey et al., 2013). The metabolic phenotype can be defined as the rates of consumption and production of the metabolites for every reaction of the metabolic model of interest in a determined biological context or environment.

Constraints are determined *a priori* based on either experimental or theoretical data like metabolomics, C^13^ labeling and measurements of consumption and production of carbon sources. An example may be the active and inactive reactions that reflect the biological state of the cell and can be determined, although indirectly, through specific transcriptional profiles (genes down or up-regulated). Another example of constraint includes the activation of transport reactions that simulates the substrate transported into the cell in a specific medium or biological condition. Therefore, the metabolic phenotype, which is defined by a set of reactions that represents a biological function, such as growth or pathogenicity, can be assessed.

Flux Balance Analysis (FBA) is an approach used in CBM to find an optimal distribution of the rates of conversion of substrates to products (fluxes), in every reaction. In order to obtain the solutions for the reaction rates of interest, a representation of a specific biological function must be defined (e.g., growth, redox potential, production of a compound of biological, or industrial interest, etc.). This specific biological function is known as the objective function. Choosing the best objective function to answer a specific biological question is still controversial. The right choice will define the robustness of the conclusions achieved by the computational analysis (see discussion below). Finally, FBA allows uncovering the most reliable mechanism behind a relevant biological function (O'Brien et al., 2013).

### Metabolic modeling of pathogenic bacteria

As mentioned above, the constraint-based modeling, CBM, has been established as a standard method for modeling the metabolism of microorganisms (especially in bacterial pathogens), given that it only relies on a few physicochemical constraints and on the assumption that the metabolic fluxes of the organism are in a steady-state (Box 3). With this approach, metabolic phenotypes of pathogenic bacteria may be simulated. Such simulations may reflect differences between wild-type bacteria and their mutant derivatives, between pathogenic and non-pathogenic bacteria, and the effect of growth at different environmental conditions.

The main biological questions addressed in metabolic models of plant pathogens, using CBM, are related to the search for control strategies against these pathogens, the classification of pathogens, the comparisons between pathogenic and non-pathogenic strains, and the plant-pathogen interactions. The CBM approach allows studying the metabolism of pathogens for the search of alternative strategies for control, and through several *in silico* and experimental approaches, has aimed to reveal the metabolic mechanisms, genes, and proteins that are important for pathogenicity. An example is the study of xanthan, a virulence factor of industrial importance, in *Xanthomonas campestris* pv. *campestris* (*Xcc*) (Schatschneider et al., 2013). Another example, includes the study of metabolic precursors of lipopolysaccharides in *Pectobacterium carotovorum* because of their role in antimicrobial resistance (Wang et al., 2014a). These two studies highlight the importance of virulence factors in the relocation of resources for pathogen growth and their potential use as drug targets.

Gene essentiality analyses have been used to find genes that are related to pathogenicity through the systematic deletion of every gene related to metabolism. The *in silico* deletion of genes in the whole reaction network allows the identification of important genes for the survival of the pathogen (Segrè et al., 2002; Shlomi et al., 2005; Kim et al., 2007). In the case of *X. campestris* pv. *campestris*, several essential genes were identified *in silico* (Schatschneider et al., 2013). Furthermore, the researchers performed experimental validation by generating mutants of the carbohydrate metabolism and xanthan production. Interestingly in this study, a subset of these genes, that were initially identified as non-essential, were found to cause a meaningful decrease in the growth rate, after additional *in silico* double mutants were performed. This highlights the importance of double mutants for the determination of essential genes and the reduction of false negative results in pathogenicity assessments.

The CBM approach has helped to compare pathogenic and non-pathogenic bacteria (Perumal et al., 2009; Charusanti et al., 2011; Liao et al., 2011; Monk et al., 2013). Correctly classifying and comparing between pathogenic and non-pathogenic bacteria is important because differences between these may help select the best target for pathogen control. Also, the CBM approach can improve our understanding of the emergence of new pathotypes and their adaptation process to different niches (Monk et al., 2013). Thus, pathogenic mechanisms and infection strategies may be revealed through CBM. However, there are also cases where the metabolic model wrongly predicts the ability of different bacterial mutants or strains to grow on different media. The reasons are metabolic reconstruction artifacts such as incomplete genome information and gaps in our knowledge of the metabolism. However, metabolic network reconciliation methods have been developed to improve the level of prediction of the models (Oberhardt et al., 2011). Ultimately, the inclusion of exact metabolic parameters such as rates of metabolic conversion and rates of volume dilution, achieved through bottom-up approaches, will improve the level of prediction of metabolic models at a genome-scale (Bruggeman and Westerhoff, 2007).

### Multiscale metabolic modeling

Several studies have focused on integrating different omics information (e.g., RNA-Seq, microarrays, metabolomics, etc.) into the metabolic, protein-protein interaction, and regulatory models of pathogens. Also, the metabolic interactions between hosts and pathogens have been subject of study. This integration has improved the phenotypic predictions, the understanding of the mechanisms of host-pathogen interactions, and have helped discover new drug targets in pathogens (Colijn et al., 2009; Bordbar et al., 2010; Ward et al., 2010; Lobel et al., 2012; Schaadt et al., 2013).

#### Control at the metabolic phenotype in bacterial pathogens

Different approaches have been proposed for integrating regulatory and metabolic models in bacterial pathogens of humans, these have not been reported so far for plant pathogens. For example, the regulatory network and the CBM model of *Mycobacterium tuberculosis* were integrated using a probabilistic approach; this model was used to predict the growth rates of different mutants and putative drug targets (Chandrasekaran and Price, 2010).

A similar approach was used in *Listeria monocytogenes* to decipher its metabolic requirements and the relationship between metabolism and virulence regulation (Lobel et al., 2012). The researchers found a correlation between the activity of certain gene regulators, under nutrient limiting conditions, and the activation of a global virulence response.

The integration of regulatory models and the metabolic model combined with experimental data is fundamental for adjusting the predictions of the metabolic phenotype. For example, Bartell and collaborators found that inconsistencies between the growth rate of *Burkholderia* in different carbon sources, that were experimentally measured, and the predictions obtained by the simulations of the metabolic model, could be partially explained by the absence of the integration between a regulatory model and a metabolic model (Bartell et al., 2014). In the previously mentioned studies, of *M. tuberculosis* and *L. monocytogenes*, researchers included in the metabolic model data obtained from experimental techniques such as microarrays, mutants, transcription factor, RT-qPCR, and lux reporters. These examples highlight the importance of experimental feedback and validation of the model for improving computational predictions, and the integration of regulatory networks into metabolic models.

A subsequent step after the coupling of the regulatory and metabolic models is the incorporation of signaling networks into the pathogenic bacterial model. For example, in *Pseudomonas aeruginosa* several genes related to quorum sensing (QS), an important process in pathogenesis that regulate the expression of virulence genes, were modeled through a multi-level approach using a Boolean method of the signaling, regulatory and metabolic networks (Schaadt et al., 2013). In this work, the researchers identified the best targets at the signaling and metabolic level to inhibit the production of auto-inducers and thus, disrupt the cellular communication between bacteria at the QS system level.

Another example of a multilevel model is *Mycoplasma genitalium* (Karr et al., 2012). This was the first effort to construct a whole model of a microorganism. In this study, 28 different submodels were used to represent the life cycle of the bacterium at the regulatory, metabolic, and signaling level. To accomplish this task, four mathematical approaches were used: (i) Ordinary differential equations, (ii) Boolean logic, (iii) probabilistic, and (iv) CBM approach.

Finally, the integration of molecular networks can be used to study microbiome interactions in pathogenic and non-pathogenic bacteria. In a study of two bacterial species, *Clostridium difficile* and *Barnesiella intestinihominis* the interaction at the metabolic level was investigated. The researchers found in their *in silico* analysis that the competition between the two bacteria reduces the growth of one of them at the expense of the other; this result was experimentally validated (Steinway et al., 2015).

#### Host-pathogen interactions

The interaction between hosts and pathogens has been widely studied in human pathogens through network biology. The research focus can be either the pathogen or the host, depending on the biological question. For example, two different studies of the interaction between *M. tuberculosis* and humans were conducted, both based on genome-scale metabolic using a CBM approach. In the first one, researchers exposed the pathogen to human macrophages, human sputum, and other *in vitro* conditions, and then integrated transcriptomics data of each condition into the metabolic model of the pathogen (Bonde et al., 2011). The objective, in this case, was to study the metabolic changes in the pathogen caused by the interaction with the host in a similar way as has been done for regulatory-metabolic networks. In this research, a down-regulation of the central metabolism and an up-regulation of the cell wall and virulence factors in the pathogen were found. In the second study, the objective was to investigate the metabolic changes in the human alveolar cells as well as in the pathogen, *M. tuberculosis* (Bordbar et al., 2010). In this study, transcriptomic data was also used to assess the interaction between the host and the pathogen. Here, the two metabolic models of both the host and the pathogen were integrated. As a result, a reduction of the metabolic plasticity of the host when interacting with the pathogen (in a technical sense: they found a reduction of the solution space of fluxes in the metabolism of the host) was found. Also, the gene essentiality analysis was improved by the incorporation of the interaction in the modeling process.

Other software tools can be used to model metabolic interactions between the host and the pathogen as are the E-flux (Colijn et al., 2009) or NetGenerator (Schulze et al., 2015) approaches. The E-Flux tool extends the genome-scale reconstructions and CBM approach, by integrating transcriptomic data into the model. Using this tool, it was possible to measure the impact of 75 drugs and nutrients on the cell wall synthesis and fatty acid biosynthesis on *M. tuberculosis*, identifying several inhibitors; importantly, some of the drugs tested are among the most widely used in the treatment of this disease (Colijn et al., 2009). The NetGenerator tool allows the incorporation of different time points in host-pathogen interactions. This method has been used to infer regulatory changes between *Candida albicans* and dendritic cells of *Mus musculus* at different time points during the interaction (Schulze et al., 2015).

Plant-pathogen interactions may also be studied through network biology. For example, host-pathogen networks have been constructed using microRNA and PPIN between *Arabidopsis thaliana* and *Xanthomonas campestris* pv. *campestris*. This study provided several potential pathways of pathogenesis (Kurubanjerdjit et al., 2012). Furthermore, the change from healthy state to disease in *A. thaliana* when infected with *Pseudomonas syringae* pv. *tomato* has been assessed (Ward et al., 2010), by integrating data from microarrays and metabolomics techniques and analyses such as: Proton Nuclear Magnetic Resonance (^1^H-NMR), Flow Injection Electrospray Mass Spectrometry (FIE-MS), Gas chromatography-mass spectrometry (GC-MS) and GC-TOF-MS (TOF by “time of flight”). This study found that the metabolism of sugars is modified in the plant to improve the flow of energy into the bacteria. Other modifications were nitrogen mobilization and purine metabolism. On the other hand, the plant showed an unusual metabolic activity of aromatic amino acids and secondary metabolites (toxins) potentially used as a defense mechanism against the pathogen.

Plant-pathogen interactions have also been modeled completely *in silico*. Duan et al. (2013) investigated five host-pathogen metabolic models. They analyze two main points: the impairment of the plant by the pathogen and the divergence between host and pathogens' networks. They calculated the metabolic impairment of the plant by identifying the metabolites from the plant that, when taken by the pathogen, affect the plant's growth (in other words, modifies the value of the objective function after FBA). The researchers found that the impairment of the plant metabolic network is determined by the pathogen and not by the host. For the comparisons between host-pathogen interactions, the authors used a multidimensional scaling (MDS) analysis. The MDS approach allows the comparison among different types of host-pathogen interactions. Using a Jaccard distance to measure the pairs of metabolic networks, authors found that the five metabolic networks of the plants studied are very similar to each other. In contrast, the pathogen networks are much more heterogeneous among them. For example, the metabolic networks of the bacterial pathogens *Xanthomonas oryzae* and *P. syringae* differed from those of the fungal pathogenic species. Additionally, researchers found that histidine is the main target in all host-pathogen interactions, followed by lysine, methionine, and the nucleotide phosphate TTP; and in the specific case of *X. oryzae*, thymidine triphosphate. They also found that the large secondary metabolism of plants is underrepresented by a gap of knowledge. However, authors recognize a bias in their study as they only compared pathogenic interactions. The solution proposed, is to use, in addition to the plant-pathogenic networks, non-pathogenic interactions as a null model to compare and validate the results found *in silico*. However, how can this *in silico* simulations be contrasted with experimental data? Interactions among non-pathogens and their host may be compared to pathogenic interactions at the metabolic level to add experimental information to *in silico* predictions.

### Objective function in pathogenic bacteria

The objective function is indispensable for the CBM approach because it specifies the set of metabolites that must be used to optimize the system and resolve the metabolic fluxes of the organism. The most frequently used objective function to model pathogenic bacteria is biomass (Table 2) (Charusanti et al., 2011; Liao et al., 2011; Thiele et al., 2011; Fong et al., 2013; Monk et al., 2013; Schatschneider et al., 2013; Wang et al., 2014a).

**Table 2:** Examples of objective functions used and the biological utility of the studies.

**Organisms**	**Biological question—objectives**	**Objective function**	**References**
*Yersinia pestis CO92*	Gene targets for antibiotic development. Growth at different carbon sources (used for classification of strains of *Y. pestis*).	Biomass: at two temperatures. Differences in LPS and fatty acid composition at biomass definition.	Charusanti et al., 2011
*Salmonella enterica* serovar Typhimurium LT2	Metabolic reconstruction, reconciliation of two models.	Biomass	Thiele et al., 2011
*Salmonella enterica* serovar Typhimurium	Reconciliation of simulations and experimental data; gap filling.	Biomass	Fong et al., 2013
*Pseudomonas putida KY2440 & P. aeruginosa PA01*	Search for drug targets and comparison of metabolic networks of pathogenic and non-pathogenic bacterium.	NA	Perumal et al., 2009
*Burkholderia cenocepacia* j2315 & *B. multivorans* ATCC 17616	Differences and similarities in pathogenesis and virulence.	Biomass: special composition of lipids and fatty acid.	Bartell et al., 2014
*Pectobacterium carotovorum* PC1	Establishes a new strategy for identification of bactericides targets of agriculture importance.	Biomass: *E. coli*	Wang et al., 2014a
*Klebsiella pneumoniae*	Metabolic model reconstruction and experimental validation of the model.	Biomass	Liao et al., 2011
*Xanthomonas campestris* pv*. campestris*	Uncover mechanisms of xanthan biosynthesis for industrial purposes and pathogenicity research.	Biomass/ xanthan production	Schatschneider et al., 2013
*Xanthomonas oryzae* pv*. oryzae & Pseudomonas syringae* pv*. tomato*	Research on plant-pathogen interactions.	NA	Duan et al., 2013
*Escherichia coli* (55 strains) & *Shigella* (8 species)	Determination of limits between strain and species at a metabolic level. Characterization of **pan** and **core** metabolic capabilities. Evaluation of strain-specific auxotrophies.	Biomass	Monk et al., 2013

When the organism under study lacks experimental data for the formulation of the biomass function, data from *Escherichia coli* is used. However, differences in the composition of biomass of the components should be considered to correct for the growth estimation of the model. For example, the biomass composition of *Klebsiella pneumoniae* has a greater proportion of carbohydrates than that of *E. coli* (probably due to differences in the polysaccharide content of its capsule); this factor was included in the model of *K. pneumoniae* and it led to an improvement in growth predictions for this species (Liao et al., 2011). Similarly, in a study performed with *Burkholderia*, it was found that the special fatty acid and lipid composition of this species was dependent on the growth temperature. Thus, this information was taken into account when determining the biomass composition used for the objective function to improve the growth predictions of this pathogenic bacteria (Bartell et al., 2014).

An important modification to the biomass function is the inclusion of the growth associated maintenance (GAM) and non-growth associated maintenance (NGAM) energies (Thiele and Palsson, 2010) as was performed in *K. pneumoniae* (Liao et al., 2011). The GAM is a reaction that represents the energy necessary (ATP) for the replication of the cell including DNA, protein, and RNA synthesis. The NGAM represents the energy necessary (also in ATP) for maintenance of the cell in activities other than growth (e.g., turgor pressure or membrane leakage). The objective is to adjust the model to the experimental growth data and to account for the differences among strains (Varma and Palsson, 1994).

Another objective function that has been used for pathogenic bacteria are virulence factors. Xanthan, in *X. campestris* pv. *campestris* (*Xcc*) was chosen with excellent results (Schatschneider et al., 2013). The main difficulty for the model of *Xcc* under the phenotype of xanthan was the lack of information in the metabolic databases regarding the polysaccharide biosynthesis needed for xanthan production. This gap was filled by Schatschneider et al. (2013) using additional information from the genome annotation performed in a former study (Vorhölter et al., 2008). Another problem detected by Schatschneider et al. (2013) was that the biomass function competes for the same precursors as xanthan. Thus, for the analysis, xanthan may be defined as a product along with biomass in a specific ratio. The most important result of this study was the discovery of an increased growth rate in the absence of xanthan production by a reallocation of carbohydrate precursors to the biomass products. Finally, the authors validated this prediction using experimental mutants of the carbohydrate metabolism and xanthan production (Schatschneider et al., 2013).

Bartell et al. (2014) extensively assessed the production of several virulence factors of *Burkholderia* species during cystic fibrosis in humans by *in vitro* and *in silico* assays. The virulence factors included biofilm-related exopolysaccharides, molecules that trigger the immune response, phagocytosis-resistant molecules, and quorum sensing molecules. The main findings from these simulations were that the most important carbon source to produce the virulence factors assessed are tyrosine and glucose and that every virulence factor can be produced by at least one carbon source. These results have been useful for drug and control design, as the specificity of the species for carbon sources was shown.

With all this taken into account, which objective function should be used for metabolic modeling? Which biomass formulation should be used? Or should it be related to pathogenesis or virulence? Or a combination of both? The final answer is in the nature of the biological question or aim to be achieved. A first approach to the model, using the biomass formulation alone, can be used to calibrate the model and assess the normal behavior in standard conditions of *in vitro* culturing. However, if a deeper understanding of the host-pathogen interaction is desired, a pathogenic/virulence focus objective function must be proposed and supported by experimental data. A final comparison between the three results of modeling with: biomass, pathogenic, and a combination of both could give insights into the pathogenic behavior. One example of the improvement of objective function based on experimental data in pathogenesis is in *Ralstonia solanacearum*, where the researchers assessed the trade-off between virulence and proliferation (Peyraud et al., 2016). Another example was proposed by researchers to modify the objective function of *M. tuberculosis* based on proteomics data, successfully improving the predictions under antibiotic stress (Montezano et al., 2015).

In conclusion, metabolic networks may be analyzed by the CBM approach without knowing all the metabolic parameters. The predictions provided by CBM can help uncover the pathogenicity mechanisms in plant pathogenic bacteria. Also, the design of control strategies against pathogens may be done by simulating multiple mutants *in silico* and then testing potential candidates in the laboratory. However, one of the weaknesses of the actual definition of objective function for metabolic studies, is the lack of experimental data to improve and confirm the predictions of the non-model organisms. Thus, unless the utility of top-down approaches for genome-scale modeling is evident, a better effort for obtaining experimental data for non-model organisms is necessary to assess the level of bias of using information of model organisms for non-model ones. Furthermore, other elements must be included in the biomass formulations as metabolic cofactors. These, have an impact in the predictions of growth of different strains on different media, as shown in previous studies (Xavier et al., 2017). Today, the CBM is the standardized approach for conducting metabolic analyses. New methods that complement CBM are being developed and incorporate regulatory, lipidomics, and transcriptomics data. This will certainly help improving the power of the predictions.

## Protein-protein interaction networks

A fundamental aspect of systems biology is the understanding of the interaction of its components in a holistic way. For networks of proteins, interactions allow the establishment of clusters and routes that proteins develop during a process (Singh et al., 2007). Each of these clusters of interactions describes a function e.g., signal transduction, assembly of the cytoskeleton, protein degradation, etc. (Zhang, 2009).

One of the great advantages that the reconstruction of PPINs provides is the ability to obtain evidence of synergy^2^, redundancy^3^, re-wiring^4^, robustness^5^, and even evolutionary processes (Sun et al., 2012). For example, the analysis of disturbance (where individual proteins are eliminated from the network) applied to a PPIN helps identify critical proteins in the system (Yadav and Babu, 2012). In addition, it is possible to integrate PPIN with other kinds of networks (for example regulatory and metabolic networks) or information to improve the understanding of microorganisms (Gligorijević and Pržulj, 2015). Finally, experimental techniques may be used to improve the reconstruction of the PPIN or to validate specific protein-protein interactions. The main experimental techniques used are shown in Table 3. A good example of the utility of high-throughput experimental techniques for PPIN reconstruction in plant pathogens is the Yeast Two-Hybrid (Y2H) system. In this study, the interaction between *A. thaliana* and three pathogens: *P. syringae, Hyaloperonospora arabidopsidis*, and *Golovinomyces orontii* (Weßling et al., 2014) were assessed. Importantly, the researchers found *Arabidopsis* target elements shared by the three pathogens, highlighting the importance of a few hubs in plants that can be targeted by pathogenicity weapons of the microorganism. This highlights the relevance of the integration of experimental techniques in pathogenicity studies.

**Table 3:** Main experimental techniques used for reconstruction or validation of protein-protein interaction networks.

**Technique**	**Large scale implementation**	**Binary interaction or complex**	**Advantage**	**Disadvantage**	**Organisms**	**References**
Y2H - Yeast two hybrid	+++	B	No antibody required	Elevated rate of false-positives; Nuclear localization of proteins	*Francisella tularensis; Blumeria Graminis; Pseudomonas syringae; Hyaloperonospora arabidopsidis; Golovinomyces orontii*	Weßling et al., 2014; Wallqvist et al., 2015; Pennington et al., 2016
PCA - Protein-fragment complementation Assay	++	C	Interaction with membrane proteins	Works better with small monomeric proteins	*Vibrio cholerae; Escherichia coli*	Ozawa et al., 2001; Hatzios et al., 2012
FRET - Förster resonance energy transfer	+	B	Reversible interaction	Decreased sensibility; Photobleaching	*Hordeum vulgare*	Bhat et al., 2005
BiFC - Bimolecular fluorescence complementation	+++	B	Used for localization in living cells	Detection of weakly associated proteins	*Agrobacterium tumefaciens*	Lacroix et al., 2005
TAP - Tandem affinity purification-mass spectroscopy	+	C	Accurate and efficient for multiprotein complex	High experimental effort and extensive data analysis	*Measles morbillivirus; Candida albicans*	Kaneko et al., 2004; Komarova et al., 2011
Protein array	++	C	Highly specific recognition	Needs a set of labeled proteins	*Staphylococcus Aureus*	Scietti et al., 2016
Pull - down	+++	C	Medium level of standardization	Protein GST fusion may cause sterical hindrance	*Streptococcus suis*	Li et al., 2016
Phage display	+++	C	Great diversity of variant proteins that can be represented in a phage library	Post-translational modifications; selection condition of library	*Helicobacter pylori*	Jonsson et al., 2004

In the following section, we will discuss some approaches for the analyses of PPIN in the context of pathogenicity interactions. The concepts used for characterizing and comparing networks were previously defined (Table 1 and Box 4).

### Computational methods in PPIN for pathogenic interaction studies

Among the multiple computational analyses that can be performed for the reconstruction of PPIN and prediction of interactions (Table 4), we will focus on phylogenetic methods, used in bacterial pathogens (Albert, 2007). We will also discuss the importance of modeling the dynamics of PPINs and how PPIN can be used for gaining insights into the meaning of pathogenicity. The reader can review other methods for PPINs reconstruction elsewhere (Dyer et al., 2007; Zahiri et al., 2013).

**Table 4:** Computational methods for prediction of protein-protein interaction.

**Technique**	**Algorithms**	**Strengths**	**Weaknesses**	**Organism**	**Reference**
Phylogenetic	Cluster analysis, maximum likelihood, maximum parsimony, Bayesian inference	Provides information of selective environmental pressure	Difficult to estimate divergence of proteins	*H. pylori, P. falciparum*	Ratmann et al., 2007
Machine learning	Random forest, decision tree, k-nearest neighbors, bayesian, Neural networks, support vector machine	Simple to understand, accurate	Dependent of parameter settings and features, black-box predictor, large data set for training	*Vibrio cholerae, P. aeruginosa*	Nanni et al., 2012; Ehrenberger et al., 2015
Data mining	Named entity recognition, ID3, Computational of natural language processing, C4.5	Fast and process large volumes of information, good to focused list	It is sensitive to noise, require manually curation	*H. pylori, Campylobacter jejuni*	Bock and Gough, 2003
Topological	*Power-law* degree distribution, clustering coefficient	Common topological characteristics among species (small-world), comparison with random networks	False positives proportional to the size of the network, configuration of protein modules may vary	*E. coli*	Butland et al., 2005; Wuchty, 2006; Sharan et al., 2007
Structure	Shape complementarity, rigid-body docking, heuristic potential	Accurate, good availability of data for primary and secondary structure	Slow development for high throughput methodologies	*E. coli, S. typhimurium* and *T. maritima*	Matsuzaki et al., 2014

#### Phylogenetic methods: orthologous domains or genes

A first methodological approach within PPIN consists of the identification of interacting proteins based on orthologous genes that are known to interact (He et al., 2008). For this approach, databases of interactions from well-characterized organisms such as *Homo sapiens, E. coli, Saccharomyces cerevisiae, Caenorhabditis elegans*, and *Drosophila melanogaster*, can be used. He et al. (2008) used these databases for the prediction of protein-protein interactions for *Magnaporthe grisea*, a pathogenic fungus that produces rice blast disease. In this study, they identified orthologous genes corresponding to proteins that are known to interact using databases from *E. coli, S. cerevisae, C. elegans, D. melanogaster*, and *H. sapiens*. They obtained a network of around 3,000 proteins for *M. grisea*. Among these, 40 seemed to be hubs that showed a high network degree. All the interactions were validated through *in silico* approaches and authors found possible pathogenic clusters involved in infection, such as phosphorus metabolism, chromatin silencing, and ion transport. This study highlights the importance of the network approach for predicting interactions where no previous information for the organism is available.

A second approach uses different protein features related to known protein-protein interactions: a motif, a domain, or a tridimensional structure. Then, these features are used to predict new interactions (Davis et al., 2007). The predictions of host-pathogen protein interactions have been mostly based on the *S. cerevisiae* interactome^6^ [which was reconstructed based on affinity purification/mass spectrometry (Collins et al., 2006)]. This interactome created a reference map which was curated for later studies. For example, Davis et al. (2007) used it to predict interactions of 10 human pathogens, including *Plasmodium* and *Mycobacterium* species, generating a full protocol based on protein domains.

In the case of plant-pathogens, a prediction at the genome-scale was calculated for *A. thaliana* and *P. syringae*. This was done through two methodologies based on domains and interolog^7^, generating more than 85,000 interactions, of which 11,000 were shared by the two methodologies (Sahu et al., 2014).

Despite the power of phylogenetic methods, they can be largely affected by the number of genomes used and the quality of their assembly and annotation. Therefore, a robust methodology of verification of false positives is necessary to evaluate the accuracy of these methods.

#### Modeling dynamic networks

Protein networks have been presented so far as a mechanism that allows associations to be viewed in a static way. In contrast, the cell performs processes precisely by receiving and emitting signals in a temporal context (Przytycka et al., 2010). The study of dynamic networks aims to identify changes in topology, function, spatial distribution, and information flow, to understand the organism's response to disturbance in function of time.

For example, probabilistic approaches integrate gene expression profiles from different time points and protein interaction data for the reconstruction of more accurate PPIN than the networks that rely only on one time point (Zhang et al., 2016). This probabilistic approach identifies protein complexes better than static methods and localizes the protein complex in their correct time stage at biological level. The authors exemplify this in the case of a protein complex of the Golgi transport system, showing their interaction in a specific point of the time series (Zhang et al., 2016).

Also, integrative strategies (using proteomics, genomics, and transcriptomics) have been generated to observe changes at the level of protein interaction or gene expression, both permanent or transient for the detection of biomarkers of disease progression as reviewed by (Wang et al., 2014b).

Temporary associations generate rapid response mechanisms, vital in defense processes against pathogens. Therefore, dynamic networks could be used for generating models of disease progression, helping in the design of drugs or control strategies (Przytycka et al., 2010).

### PPIN in the context of host-pathogen interactions

We want to point the main use of PPIN approaches in pathogenicity context. First, multiple protein-protein interactions among pathogenicity factors (e.g., effector proteins) and host proteins (based on genome data and information of related species) can be assessed *in silico*. Then, the target of the protein of the pathogen into the host can be predicted, and obtain a network of PPIN of the pathogen and the host. Second, host-pathogen interactions can be assessed through techniques as Y2H or other techniques mentioned at the beginning of this chapter. Then, these experimental data and *in silico* predictions can be used to construct a PPIN of the host-pathogen interaction. This kind of network has a lot of information useful for the biotechnological control of the pathogen. For example, with the help of network metrics (Table 1) such as clustering coefficients or network degree, hubs of susceptibility in the host can be detected. Finally, it is highly recommended to experimentally confirm the candidates by more precise techniques such as CoIP.

### Signaling networks: a special case of PPIN

Signaling is a series of chemical and/or energetic transmissions from an external stimulus to the cell. Signaling networks reconstruct the interaction path of the signal-carrying elements (usually proteins) to the organelle that requires the decision of maintaining or changing a state of homeostasis (Cho et al., 2015). These networks are also represented in a directed manner (Figure 1A) and with highly conserved and specific topologies. Usually, these types of networks also include transcription factors and PPIN to reconstruct the signaling cascade. The most likely edges of signal conduction are also weighted by the strongest or most reliable directed path (Cho et al., 2015). From the computational point of view, the weighting of the vertices constitutes a great challenge due to the inherent subjectivity of this process. Punctuation methodologies have been proposed by defining a probability (Liu and Zhao, 2004).

Kim et al. (2014) discuss the robustness and modularity of an immunity network, specifically that of *A. thaliana* under a pathogen attack, investigating the changes of the plant-immunity process called pattern-triggered immunity. They constructed a dynamic model of a signaling network by evaluating the determination of certain plant hormones against the immune challenge, to evaluate their predictive power. They found that the hormone ethylene increases the robustness of the system by inhibiting the jasmonate pathway. With this, they could conclude that the network is able to grade the level of the response to a given pathogen.

## Regulatory networks

Regulatory networks represent the relationship between genes and regulatory proteins that lead to the expression or suppression of certain genes. The graphs of regulatory networks are represented in a directed way (Figure 1A), trying to capture a series of events that are often consecutive. These networks show highly defined and sometimes hierarchical modules (Lozada-Chávez et al., 2006).

Regulatory networks are highly dependent on the environmental conditions, the cell type that is being studied, and the developmental stages of the organism. Due to the nature of this type of networks, mechanisms of control and modulation generally given by transcription factors need to be considered (Lee, 2002). Moreover, these networks may represent protein-DNA interaction. Thus, they may be easily integrated into protein-interaction networks and metabolic networks.

Because of the large amount of information that is possible to integrate to these networks, multiple approaches have been implemented, based on different sources of information (Marbach et al., 2012). Table 5 summarize some of the methods used for the reconstruction of regulatory networks. For instance, in a report on the plant pathogen *Xanthomonas axonopodis* pv. *citri*, researchers used microarrays and mutants to decipher the role of two proteins, HrpX and HrpG, in the global control of the virulence process (Guo et al., 2011) and proposed a regulatory model. Also, Seo and collaborators used analysis of gene expression profiles and ChIP-chip experiments to uncover the main transcriptional architecture and regulatory features of *K. pneumoniae* (Seo et al., 2012).

**Table 5:** Methods for reconstruction of regulatory networks.

**Approaches**	**Highlights**	**Challenges**	**Organisms studied**	**References**
Differential equations	Network dynamic over time, regulation and optimization of function	High computational demanding, complex parameter optimization	*Mus musculus, Candida albicans*	Linde et al., 2015
Boolean	Switch-like behavior, efficient and easy interpretation	Only two states, good in small networks, Only synchronous interactions	*H. pylori*	Franke et al., 2008
Bayesian^*^	Robust to deal of disturbances, integrated knowledge to increase the support	Non-dynamical, high computational cost, often used a hybrid method to increase the accuracy	*E. coli*	Yang et al., 2011
Neural networks	Allows continuous variables over time, very sensitive for regulated systems, noise-resistant	Computational complex, difficult for training, need a lot of input data	*Caulobacter crescentus, E. coli, Bacillus subtilis*	Yaghoobi et al., 2012; Umarov and Solovyev, 2017
State space model	High computational efficiency, probabilistic framework to simulate the network, determines an optimal threshold value	There are no learning steps	*Saccharomyces cerevisiae, Aspergillus fumigatu*	Do et al., 2009; Koh et al., 2009

* *To counteract the stationary problem of Bayesian networks, The dynamic Bayesian network approach was developed*.

Finally, transcriptional reprogramming is a mechanism of great importance in the control of pathogenicity. Consequently, the reconstruction of regulatory networks derived from temporal series of gene expression data, available in public repositories (Marbach et al., 2012), could be used to predict the response of the pathogen to host defense or antibiotic treatment. Adding promoter regions and functional annotations can help improve this type of network and highlight key components in pathogenicity and evolution of resistance.

## Networks, evolution, and pathogenicity

### Evolution of network topology and distribution of fluxes

The comparative analysis of networks is a powerful tool that allows understanding the evolutionary relationships among organisms. Furthermore, it allows scientists to decipher the evolution of cell processes such as pathogenicity and adaptation to life on a host. In the context of metabolic networks, three main characteristics can be compared: the similarity of their components, their topology or organization, and the distribution of fluxes. Some studies that are reviewed here show several principles of the evolution of networks in pathogenic bacteria. We would like to highlight two of them: (i) highly connected elements of the network are highly conserved and (ii) in a changing environment, the organism will favor one functional objective at the expense of others.

As stated in the first principle mentioned above, the organization of the networks reflects the evolutionary conservation of its components. Some studies have shown the positive correlation between connectivity of proteins and their degree of conservation (Butland et al., 2005). The organization of the core (shared pathways) and the specific networks are related to the lifestyle of the organism. Regardless of the pathway, the highly-connected enzymes or other elements (regulatory modules and protein interactions) in the network are highly conserved. Furthermore, a scale-free network is vulnerable to the removal of the highly-connected proteins (hubs) but not to the deletion of the less connected proteins. The modularity of the networks reflects the lifestyles of the organisms, as will be discussed in the next section (Butland et al., 2005; Kreimer et al., 2008).

Concerning the second principle, while today we have a better understanding of the way networks are organized or their topology, the evolution and the distribution of fluxes through metabolism have been less studied. Schuetz et al. (2012) compared the evolution of metabolism in microorganisms to the Pareto optimality. The Pareto optimality or Pareto efficiency is an economic concept stating that one's utility will increase only if someone else's utility diminishes (Sen, 1993). Therefore, in a changing environment, an organism faces a series of trade-offs; the optimality of an objective will be at the expense of another (ATP balance, growth rate, or minimization of fluxes; Schuetz et al., 2012). For example, an organism cannot be optimally adapted to growth in aerobic conditions and anaerobic at the same time. More importantly, authors found a deviance of the metabolism's operation of some mutants from the Pareto surface, which support the author's hypothesis that organisms maintain some space from optimality as evolutionary adaptation under changing environments (Schuetz et al., 2012). Thus, evolution favors flux distributions that minimize adjustments to the new conditions (Schuetz et al., 2012).

### Comparative studies of networks

Network comparisons between different organisms to study their evolution can be performed with different methods. Some methods compare the contents of the network (e.g., similarity in enzymes, individual pathways, or the whole repertoire) while others compare their structure. We will revise some of these methods mentioning their differences and some of their applications.

The first set of methods calculates indices of similarity or distance between networks, by calculating the similarity or distance between the network components (enzymes, transcription factors, or any other sequence used to construct the network). The similarity between proteins can be simply obtained by their sequence or structure similarity but also by the similarity between the EC (Enzyme Classification) numbers of the corresponding reactions, in the case of metabolic networks (IUBMB. Nomenclature Committee of The International Union of Biochemistry and Molecular Biology, 1992; Heymans and Singh, 2003).

Other methods use the information of the structure of the networks. Forst and Schulten (2001) used sequence similarity combined with information of the corresponding network. They defined the distance between pathways based on all the comprising elements that share the same functional role. In the simplest pathway, the elements of a functional role are the enzyme and its substrate and they can be compared by traditional sequence comparison analysis, if the latter is a protein.

Heymans and Singh (2003) proposed to combine both measures, similarity of the components and network structure using local graph similarity. The graph similarity is calculated on enzyme subsets where all the information contained within the pathways, except for the enzymes, is deleted and the simplified subset is then compared (Heymans and Singh, 2003). However, this method applies to individual pathways and a more inclusive approach was proposed by Forst et al. (2006) in a study where the whole metabolic networks are compared (Heymans and Singh, 2003; Forst et al., 2006).

The fourth set of analyses studies differences in the components of the networks; basically, they compare the insertion or deletion of components in a network. These approaches allow the understanding of the adaptation of organisms to new niches. In a network, two types of pathways can be identified, the essential, present in all organisms, and the non-essential, which are under continuous evolution and are specific to the organism's lifestyle (Mithani et al., 2010). In the Reaction correlation analysis (Mithani et al., 2010, 2011) a Euclidean distance is calculated based on the absence or presence of the reactions in different individuals or strains. In the “all but one analysis” included in the software Rahnuma (Mithani et al., 2009), and then redefined by Mithani et al. (2010), the user can identify pathways and reactions present in some organisms but absent in others. The identification of a core network leads to the construction of an Ancestral Network, a network comprising the reactions present in all species and the definition of species-specific networks (Mithani et al., 2010). Therefore, a Bayesian model like the one proposed by Mithani et al. (2010) for the study of network evolution allows for the identification of regions of the network under selective pressures, most probably involved in pathogenicity processes. In a study combining different approaches of network evolution analysis, Mithani et al. (2011) showed how these comparative analyses lead to the understanding of the evolution and adaptation strategies of a set of related organisms, some pathogenic and other nonpathogenic. For example, according to the ancestral network reconstruction, it has been suggested that the ancestral pseudomonad was saprotrophic from which more specialized pathogens evolved (Mithani et al., 2010, 2011).

The fifth set of analyses compare the topological features of metabolic networks, especially modularity, for more than 300 bacterial species (Kreimer et al., 2008). These analyses permit studying evolution at a broader phylogenetic scale and relate network characteristics with environmental cues. One of the main results of Kreimer and collaborators was that the environmental factors influence network modularity. Also, symbionts and pathogens show lower modularity while the free-living and less niche-specific bacteria show higher modularity (Kreimer et al., 2008). Moreover, endosymbiotic organisms living in nutrient-restricted niches show both smaller networks and less modularity, losing specific fast-evolving pathways (Kreimer et al., 2008). In this context, modularity is interpreted as subset of functionally related and highly connected reactions or pathways. Thus, pathogens and symbionts have a lower number of modules and connections because they are expected to use the external pathways from the host for its own benefit.

### Network evolution and pathogenicity

The comparative genomic studies reviewed here take advantage of a higher order of organization based on the structure and properties of the molecular level network-based models. These models allow stating additional hypotheses for the evolution of bacterial pathogens. However, studies based on molecular network models have important limitations as do other comparative genomic studies. Missing data is probably the major drawback, for example on the directionality and kinetic parameters of the reactions.

The study of network evolution will help in the understanding of pathogenicity and in the processes of adaptation of pathogens to new or old hosts. Especially, the organization of orphan genes, the species-specific or pathogen-specific genes, and their connections to the core network will help achieve this goal. New genes arise by different processes: exon shuffling, gene duplication, retrotransposition, mobile elements, lateral gene transfer, *de novo*, and a combination of these mechanisms (Long et al., 2003). Once generated, both duplicated and novel genes are less connected at the beginning, however, the rewiring process differs between these two (Capra et al., 2010). In the case of the pathogenicity-related genes, it is argued that they will always occupy peripheral positions in the networks (Kholodenko et al., 2012), a result expected due to their fast-evolving rates.

The study of the rewiring process of recently evolving genes may be helpful in the pathogenicity studies, given that the rewiring process occurs not only inside the cell but also with its interactors (host or pathogen). In a recent study, it was shown that effector proteins from phylogenetically distant organisms converge to and target highly connected hubs of the immune plant system (Mukhtar et al., 2011; Kholodenko et al., 2012). Thus, this mechanism of host-pathogen interaction could help in the prediction of evolving paths in the pathogen as response to drug or pesticide control (in human and plant pathogens respectively), and therefore partially solve the problem of resistance in pathogens subject of pathogenicity control.

## Conclusions

We have reviewed the metabolic, protein-protein and regulatory networks that have helped understanding disease, mechanisms of pathogenesis and virulence, as well as interactions between bacteria and their hosts.

All types of networks, used for prediction purposes, have both strengths and weaknesses, and provide different types of biological information Table 6. Also, we showed how topological and other mathematical approaches can be used to analyze every type of network. For example, CBM, which does not rely on the complete knowledge of the kinetic constants, serves as a useful approach for metabolic analyses in pathogenic bacteria. In contrast, the Boolean analysis of regulatory networks, which relies only on topological features of the network architecture, provides useful information about pathogenic mechanisms. Thus, the different mechanisms of pathogenicity, disease, and virulence can be uncovered by network approaches. However, a strong feedback between the information derived from experimental procedures and computational models should be progressively more relevant and important to improve the conclusions of the models and provide new biological hypotheses.

**Table 6:** Summary of networks for the study of host-pathogen interaction.

**Networks**	**Experimental data**	**Mathematical and computational approaches**	**Objective**
Regulatory	Genomics; Transcriptomics; Transcription Start Site (5′-RACE); Binding sites global regulators (ChIP-chip)	Boolean; network analysis	Dynamic of regulation of genes involved in virulence and pathogenicity
Metabolic	Genomics; transcriptomics; Metabolomics; Phenotype microarrays; C13 labeling	Constraint-based modeling; elementary flux mode analysis; pathway enrichment analysis; network analysis	Metabolic capabilities; genes related with virulence and pathogenicity
Protein-protein interaction	Y2H; PCA; BiFC; Protein arrays; Pull down; Phage display	Phylogenetic methods; dynamical networks; machine learning	Identification of hubs involved in virulence and pathogenicity; Determination of interaction between proteins related with signaling and regulatory cascades
Signaling and regulatory	Transcriptomics; Fusion assays (LacZ reporter); Adherence assay; Biofilm formation (fluorescence)	Boolean; network analysis	Impact of sensors in regulation of virulence and pathogenesis; Cell-to-cell signaling; biofilm synthesis;
Signaling, regulatory and metabolic	Genomics; metabolomics; transcriptomics	Constraint-based modeling; boolean model hierarchical layers; network analysis	Model regulatory and metabolic network of QS system

*The networks reviewed in this work, the experimental data (mainly at the level of omics), the mathematical and computational approaches applied for every network, and the research objective for the networks studied are summarized*.

The systems biology approach can be used to design control strategies of the pathogen. For example, bactericides target important regulators or proteins of the pathogen, identified on *in silico* studies. In the case of regulatory networks, two of the most important aspects related to pathogens are the robustness of the network to random changes and its stability through time. This has been made evident by the high degree of fitness that successful pathogens possess. Pathogens share elements linked to pathogenicity that have simultaneous and/or complementary actions as redundant mechanisms in the event of detection by the host. The robustness is a consequence of the wired redundancy of the gene-regulator interactions, especially in the genes encoding for hub proteins. It can be inferred that the evolutionary forces have shaped and constrained the most important regulatory pathways involved in disease, pathogenicity, and virulence of bacteria. Therefore, genes within pathways that improve the fitness of the pathogen are positively selected, increasing the degree of wiring of these specific mechanisms. These genes are promising targets for bacterial control.

In the case of protein-protein interactions, new methodologies and approaches have emerged from structural, functional and computational knowledge. Studies have focused on the functional role of proteins in disease-related processes, significantly contributing to the understanding of the role of proteins as mechanistic executors in each of the physiological stages of infection. Thus, signaling pathways or hubs that are susceptible to be blocked to prevent the development of a given disease could be detected and be used to design control strategies of the pathogen. One of these strategies starts from the analysis of domains or contact surfaces allowing to establish interactomes *in silico* and develop mimetic or decoy proteins.

We have shown that regulatory, metabolic, and protein-protein interaction network systems are tightly interconnected, and each of them depends on the others. In the future, we expect that more studies center their efforts into coupled systems using different computational and mathematical approaches with the support of several experimental techniques and approaches (as much targeted to specific genes and mechanisms as supporting high-throughput data analysis). For example, the gene essentiality analysis is important in the context of regulatory networks, where deletion of genes impact molecular networks at the level of protein interactions, signaling cascades, and the metabolic phenotype. Therefore, this analysis constitutes a powerful approach for searching for genetic targets for the design of control strategies against pathogens.

Another example of the inference power of coupled systems is the relationship between the genotype and the phenotype that is reflected in metabolic and protein networks linked to regulatory and signaling networks. It is the convergence of systems, through the switching of the distinct metabolic pathways mediated by regulation of the genes and signaling cascades, that determines the defense and attack mechanisms of the pathogen. The hubs at the level of the regulatory system play an important role in the control of pathogenicity, since a global regulator of pathogenicity can control several genes within a pathogenicity module. Subsequently, the downstream cascade of genes can up or down-regulate several other genes involved in metabolism and other functions. The result is the expression of a metabolic phenotype that serves as a coordinated attack or defense system. Thus, the study of regulatory, signaling and metabolic interactions through a multiscale modeling approach will provide promising results related to pathogenicity and defense mechanisms.

In systems biology, we will see an important improvement of the evolutionary analyses performed on the networks. The incorporation of a genetic population frame is urgently needed to help to understand the pathogenic mechanisms of host-pathogen interactions. A way to accomplish this is through the establishment of relationships between genetic variation of the genes associated with the enzymes and proteins and the properties of the networks to explain this variation in spatial and evolutionary terms in a system context. Ultimately, the host-pathogen relationships are governed by evolutionary forces acting in time and space of the whole biological system.

The evolutionary studies supported by systems biology can help to solve important questions related to pathogenicity as the emergence of specific pathogens and their relationship with non-pathogens. The processes of interaction among species over millions of years have largely been influenced by domestication. This has generated changes among the connections of the elements of the immune system (rewiring). As a result, selection pressures have varied, favoring, in some cases, a non-specific pathogen to infect a given host. This process can be modeled through networks, by reconstructing the routes or proteins of ancestral and/or non-domesticated species and comparing with the present ones to observe the changes in connections among the elements.

From the evolutionary point of view, networks can also demonstrate the molecular changes that have occurred during pathogen interactions. From the hypothesis of arms race processes, new perspectives have been generated that can fill the gaps, such as that proposed by Cook et al. (2015), which provides a view of the host-pathogen interaction, related to mutualism and parasitic symbiosis as initial stages of co-evolution. With the above, we could rethink the approximation strategies and how we understand the interaction of what is considered pathogenic, and how biological networks can drive to new hypothesis through the integration of enormous amount of information.

Finally, the comparisons between pathogens and non-pathogens in an evolutionary context, where there are conserved and divergent features among the different strains and species, can serve to design control strategies and to help to improve the understanding of pathogenicity mechanisms.

In this review, we tried to describe different methodologies to solve biological questions using the networks, giving an overview of the available mathematical approaches. As a growing discipline, network analysis in systems biology still has challenges that must be overcome and must be considered when generating new hypotheses. Some of the challenges that need to be addressed are:

- At the metabolic level, the objective function should be redefined in a context of host-pathogen relationships (xanthan is a good example; other pathogenic factors can be modeled in the same way).
- Protein-protein interaction prediction methodologies must have a large amount of data as a basis for prediction.
- The reconstruction of the regulatory networks still represents experimental limitations since a high amount of data are needed such as time series, gene deletions or biological samples.
- The evolutionary forces acting on the networks should be mathematical and computational implemented; not only to compare between different networks of the same species or genus, but also to differentiate among genetic drift, genetic flow and other evolutionary forces.
- The experimental information on non-model pathogens, especially the high-throughput data must be increased for feeding the computational models and for comparison purposes.

Confronting these challenges will bring the study of pathogenic mechanisms and relationships to a next level. Without doubt, network analysis in systems biology will appear as an essential discipline used in every molecular laboratory that studies host-pathogen interactions and, we will see a burst of user-friendly software in network biology designed for experimental biologist to fulfill this necessity.

## Author contributions

DB, CA, AB, GD, SR: Developed and wrote the manuscript; GD, SR, AB: Guided and assisted in writing the manuscript; All authors read and approved the final manuscript.

### Conflict of interest statement

The authors declare that the research was conducted in the absence of any commercial or financial relationships that could be construed as a potential conflict of interest.

## Footnotes

^1^The metabolic phenotype is the distribution of the biochemical reactions rates (fluxes) in a determined set of conditions (physicochemical constraints).

^2^**Synergy**: union of two or more processes or paths that generate new process or biological properties.

^3^**Redundancy**: repetition of process or elements that serve as a functional reserve in case of failure.

^4^**Rewiring**: change in the association of biological entities that can vary along the time for improvement of system efficiency.

^5^**Robustness**: capacity of the biological system to recover from perturbations conserving the equilibrium of the system.

^6^It is appropriate to clarify that the interactome includes the set of interactions that can occur in an organism, usually but not always, represented by protein-protein interaction.

^7^Interactions that are conserved among pairs of proteins that are present in descent-related species.
